# Clinical aspects of pancreatogenic diabetes secondary to hereditary pancreatitis

**DOI:** 10.1186/s13098-017-0203-7

**Published:** 2017-01-13

**Authors:** Marcio Garrison Dytz, Pedro Arthur Hamamoto Marcelino, Olga de Castro Santos, Lenita Zajdenverg, Flavia Lucia Conceição, Tânia Maria Ortiga-Carvalho, Melanie Rodacki

**Affiliations:** 1 Endocrinology Section, Department of Internal Medicine, Medical School, Universidade Federal do Rio de Janeiro (UFRJ), Rio de Janeiro, Brazil; 2 Laboratory of Translational Endocrinology, Instituto de Biofísica Carlos Chagas Filho, Universidade Federal do Rio de Janeiro (UFRJ), Rio de Janeiro, Brazil; 3 Diabetes and Nutrology Section, Department of Internal Medicine, Medical School, Universidade Federal do Rio de Janeiro (UFRJ), Rio de Janeiro, Brazil; 4 Endocrinology Section, Hospital Universitário Clementino Fraga Filho, Rua Rodolpho Paulo Rocco 255, Ilha do Fundão, Rio de Janeiro, RJ 21941-913 Brazil

**Keywords:** Hereditary pancreatitis, Cationic trypsinogen, Pancreatogenic diabetes, Beta-cell

## Abstract

**Background:**

Hereditary pancreatitis is a rare inherited form of pancreatitis, characterized by recurrent episodes of acute pancreatitis with early onset and/or chronic pancreatitis, and presenting brittle diabetes, composed of episodes of nonketotic hyperglycemia and severe hypoglycemia. The existing literature regarding this form of diabetes is scarce. In this report, clinical features of pancreatogenic diabetes secondary to hereditary pancreatitis are presented along with recommendations for appropriate medical treatment.

**Results:**

Clinical data from five patients of a family with pancreatogenic diabetes secondary to hereditary pancreatitis were analyzed. The average time between hereditary pancreatitis and diabetes diagnosis was 80 ± 24 months (range: 60–180 months) with a mean age of 25.6 ± 14.7 years (range: 8–42 years), four patients used antidiabetic agents for 46 ± 45 months and all progressed to insulin therapy with a mean dose of 0.71 ± 0.63 IU/kg (range: 0.3–1.76 IU/kg). The glycemic control had a high variability with average capillary blood glucose of 217.00 ± 69.44 mg/dl (range: 145–306 mg/dl) and the average HbA1c was 9.9 ± 1.9% (range: 7.6–11.6%). No ketoacidosis episodes occurred and there were several episodes of hospitalization for severe hypoglycemia.

**Conclusions:**

Diabetes mellitus secondary to hereditary pancreatitis presents with early onset, diverse clinical presentation and with extremely labile glycemic control. Diabetes treatment varies according to the presentation and insulin is frequently necessary for glycemic control.

## Background

Hereditary pancreatitis (HP) is a rare autosomal dominant disease characterized by recurrent episodes of acute pancreatitis that leads to permanent chronic pancreatitis. Common clinical manifestations are: abdominal pain, disabsorptive syndrome, diabetes mellitus (DM) and pancreatic cancer [1].

Pancreatic inflammation results in destruction of pancreatic islet with loss of β-cells (insulin), α-cells (glucagon), δ-cells (somatostatin) and PP-cells (pancreatic-polypeptide) [2] that can lead to the development of brittle diabetes, which is characterized by extreme blood glucose levels fluctuations, causing hyperglycemia or hypoglycemia [3]. This article focuses on clinical features and treatment of pancreatogenic diabetes secondary to HP.

## Methods

Clinical data from a family with confirmed molecular diagnosis of HP was evaluated. A retrospective analysis of their medical records assessed weight, age, DM duration, interval between the diagnosis of HP and DM, use and dose of insulin, use of oral antidiabetic medications, HbA1c values, episodes of severe hypoglycemia, hospitalization for ketoacidosis and presence of chronic complications of DM. Download of data obtained through thirty days of self-monitoring of blood glucose using the Accu-Check Smart Pix Software®, was performed in order to measure and calculate average and standard deviation (SD) of capillary blood glucose (CBG). The mutation screening has been previously described [4].

The local ethics committee approved the study protocol (169 11-CEP), in accordance with institutional ethical standards and national research committee. Patient informed consent form was obtained before initiating the study.

## Results

**Fig. 1:**
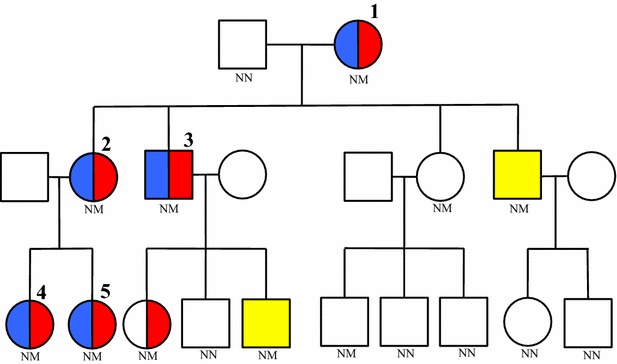
Pedigree of the reported family demonstrating 5 members with diabetes (*blue*) and exocrine pancreatic dysfunction (*red*), 1 member with only exocrine pancreatic dysfunction, 2 members with acute pancreatitis (*yellow*) and 2 clinically unaffected mutation carrier. *NN* no mutation, *NM* heterozygous mutation

The study evaluated five patients from a family with HP secondary to N29T mutation in exon 2 of the *PRSS1* gene (Fig. 1) [4]. In 3 patients, the HP diagnosis preceded DM, while in 2 the opposite occurred. The average time between HP and DM diagnosis was 80 ± 24 months (range: 60–180 months) (Table 1).

**Table 1:** Clinical features of pancreatogenic diabetes in affected patients

Patient	Age of onset (years)	Duration of pancreatitis (years)	Duration of diabetes (years)	BMI (kg/m^2^)	Mean CBG/SD	Insulin dose (IU/kg)	HbA1c^a^ (mean) (%)	Chronic diabetes complications
1	8	53	19	25.4	184/94	0.3	7.6	No
2	5	36	13	21.7	233/104.4	0.83	11.6	Retinopathy
3	14	25	2	29.7	–	0.27	8.2	No
4	2	16	10	22.6	145/94.8	0.41	11.5	No
5	4	12	5	22.7	306/127.5	1.76	10.9	Neuropathy

*BMI* body mass index,* CBG* capillary blood glucose,* SD* standard deviation

^a^Mean of 5 years, method high-performance liquid chromatography (HPLC)

All patients used insulin. The mean dose was 0.71 ± 0.63 IU/kg (range: 0.27–1.76 IU/kg). In 4 patients, other drugs (Metformin and Glyburide) were used before insulin therapy was started, mean time of 46 ± 45 months (range: 4–96 months).

**Fig. 2:**
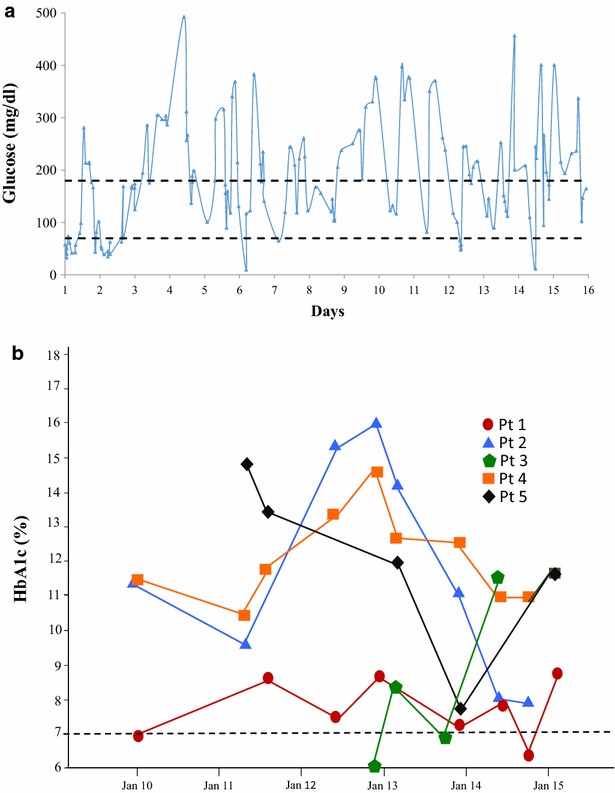
**a** Capillary blood glucose (CBG) of the patient 4 in 2 weeks with high glycemic variability. **b** HbA1c measurements of patients with Hereditary Pancreatitis over 5 years

The average CBG was 217.00 ± 69.44 mg/dl (range: 145–306 mg/dl) with SD of 104.75 ± 15.56 mg/dl (range: 94–127 mg/dl). There was a high variability of CBG in all cases, with frequent hypoglycemic events and hyperglycemic excursions. HbA1c levels demonstrated the heterogeneity between patients, but most patients showed elevated levels (Fig. 2).

The average diabetes duration was 120.80 ± 80.32 months (range: 24–228 months). No ketoacidosis episodes occurred, although there were episodes of hospitalization because of severe hypoglycemia. Surgical procedures were performed on patients 4 and 5, respectively, for refractory pain and abdominal complication. Patients 2 and 5 presented microvascular lesions secondary to diabetes with nonproliferative diabetic retinopathy with macular edema and distal symmetrical sensorimotor polyneuropathy accompanied by autonomic neuropathy, respectively.

## Discussion

In this study, we describe the glycemic pattern of five patients with diabetes secondary to a mutation in the *PRSS1* gene, which leads to an increased autocatalytic conversion of trypsinogen to active trypsin, that results in autodigestion and damage to the acinar cells [5, 6].

**Fig. 3:**
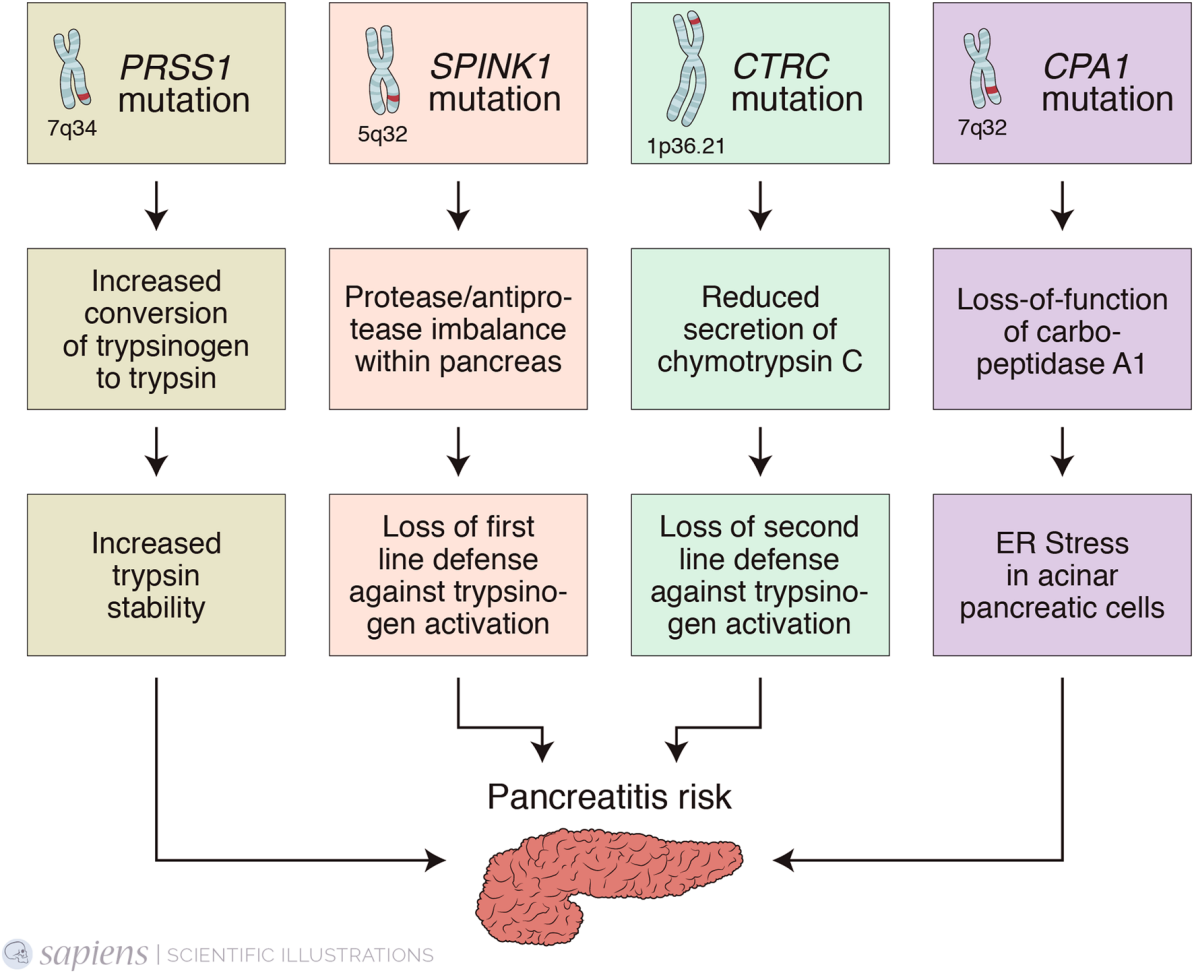
Schematic mechanism underling mutations-associated pancreatitis. The *PRSS1* (Cationic Trypsinogen) mutation leads to a gain-of-function with an increased conversion of intrapancreatic trypsinogen to trypsin. The *SPINK1* (Serine Protease Inhibitor Kazal type 1) and *CTRC* (Chymotrypsin C) mutations lead to loss of defenses against the activation of trypsinogen. Mutations in *CPA1* (carboxypeptidase A1) generate misfolded proteins leading to endoplasmic reticulum stress

Mutations in the *PRSS1* gene (R122H, N29I, A16V, and other less prevalent) are responsible for greater than 70% of mutations in HP kindreds [7, 8], but other mutations have also been described (Fig. 3) [9–11].

A high rate of hypoglycemia and CBG variability was observed in these patients with diabetes secondary to HP. The marked glycemic lability is probably due not only to a continued loss of insulin secreting β-cells but also from counter regulatory glucagon secreting α-cells. Additionally, nutrients malabsorption resulted in impaired incretin secretion and thereby diminished insulin release. Moreover, the lack of other paracrine or endocrine factors secreted by the pancreatic cells may also contribute to this glycemic pattern [12].

Unlike type 1 DM, even with high glucose levels no patient developed ketoacidosis. The reasons that may warrant this is that the β-cell deficit is seldom absolute and it occurs concomitantly with the loss of α-cells [13].

All patients required insulin therapy, but the progression of the pancreatic endocrine failure was extremely variable. In some of the cases, insulin was necessary shortly after the diabetes diagnosis, while in others, oral drug therapy without insulin was possible for years.

Oral antidiabetic agents may be appropriate in early pancreatogenic diabetes (HbA1c < 8.0%). Metformin should be the drug of first choice, in the absence of contraindications, especially if concomitant insulin resistance is evidenced, and if it is tolerated due to common gastrointestinal adverse effects and weight loss [14]. Besides that, it is possible that Metformin might reduce the risk of pancreatic cancer, and therefore would have a theoretical rationale in chronic pancreatitis [15]. Oral therapy with insulin secretagogues (sulfonylurea and glinides) may also be considered as second-line therapy, but should be aware of hypoglycemia in patients with inconsistent meal ingestion [3]. Thiazolidinediones, incretin based therapies and SGLT2 inhibitors should be avoided because of side effects and lack of data in this context [3, 16].

In advanced pancreatogenic diabetes, insulin therapy is the preferred treatment, and especially during acute episodes of pancreatitis or hospitalized patients, and severe malnutrition patients in which the anabolic effects of insulin are desired. Patients should be treated using general insulin dosing and regimen guidelines for type 1 diabetes [3, 16]. The patients studied used NPH and regular insulin because it is the first choice in the local healthcare public system. Despite the use of human insulin, glycemic control was more unpredictable than expected with high glycemic variability. The insulin analogues or insulin pump therapy are treatment options that may allow a more stable glycemic control.

Moreover, the islet autotransplant might be an option for patients with severe chronic pancreatitis, who have debilitating abdominal pain refractory to medical or endoscopic interventions and for which total pancreatectomy is indicated [17, 18]. Patient 4 underwent pancreatectomy and could have benefited from this therapy to preserve the function of the remaining β-cells.

In summary, these data indicate that DM secondary to HP presents clinical heterogeneity among patients, but with high glycemic variability and difficult management. Hence, molecular diagnosis should be performed in suspicious patients of HP because it allows the proper approach. Strategies to improve the glycemic control in affected patients should also be pursued.

## Abbreviations

HP: hereditary pancreatitis
DM: diabetes mellitus
SD: standard deviation
CBG: capillary blood glucose
NN: no mutation
NM: heterozygous mutation
BMI: body mass index
HPLC: high-performance liquid chromatography
*PRSS1*: cationic trypsinogen
*SPINK1*: Serine Protease Inhibitor Kazal type 1
*CTRC*: Chymotrypsin C
*CPA1*: carboxypeptidase A1
